# Co‐adapting the DELIGHT (Dementia Lifestyle Intervention for Getting Healthy Together) program with and for the Chinese community

**DOI:** 10.1002/alz70858_106673

**Published:** 2025-12-26

**Authors:** Liu Grace, Heather Keller, Carrie A McAiney, Kassie Harker, Dinh E Christopher, Wai Hin Chan, Carole Chow, Ho Mabel, Angela Liu, Sheng Han Liu, Cecilia Lui, Jasmine Mah, Jaqueline Vong, Olivia Vong, Anisa Wu, Laura E. Middleton

**Affiliations:** ^1^ University of Waterloo, Waterloo, ON, Canada; ^2^ Schlegel‐UW Research Institute for Aging, Waterloo, ON, Canada; ^3^ Yee Hong Centre for Geriatric Care, Toronto, ON, Canada; ^4^ Bruyère Health Research Institute, Ottawa, ON, Canada; ^5^ Rogers Television, Waterloo, ON, Canada; ^6^ ILIA Connect, Hamilton, ON, Canada; ^7^ McMaster University, Hamilton, ON, Canada; ^8^ Dalhousie University, Halifax, NS, Canada; ^9^ Playology Intl, Toronto, ON, Canada

## Abstract

**Background:**

The DELIGHT (Dementia Lifestyle Intervention for Getting Healthy Together) program was co‐designed with people living with dementia, care partners, health/social care providers, and researchers to support the health and wellness of people living with dementia and care partners. DELIGHT is an 8‐week program that includes exercise and shared learning related to healthy eating, sleep quality, social connection, mental wellbeing, and physical activity. There is a need to adapt the program to meet the needs of diverse ethno‐cultural communities in Canada.

**Method:**

We assembled a Chinese Co‐adaptation Team with eight members who were part of the Chinese community, including Cantonese and Mandarin speakers with various perspectives (i.e., person living with dementia, care partners, health/social care providers) and six researchers/project manager. Meetings were held mainly via Zoom using an authentic partnership approach with an aim to tailor DELIGHT materials to ensure relevancy for the Chinese community.

**Result:**

The team met inperson once with ten virtual meetings to co‐adapt the DELIGHT materials (Table 1). For the physical activity and sleep sections, there were few content changes. However, when discussing the resources for emotional well‐being and social connection, the Team shared insights regarding widely held stigma of dementia and mental health. For example, dementia directly translates into “crazy” in the Chinese language. Due to filial piety in the Chinese culture, adult children often experience caregiver burden, rather than empowering their elderly parents. The Team then suggested developing three new resources on understanding dementia and altered the language from mental to emotional well‐being. For the healthy eating resources, the Team provided preferred Chinese food choices and ingredients and, under the supervision of a research team member and Registered Dietitian, a Chinese Undergraduate Research Assistant created 15 brain‐healthy recipes for the Chinese DELIGHT offerings.

**Conclusion:**

The Team co‐adapted 27 resources, created 6 new factsheets and 15 Chinese recipes. Through the co‐adaption process, we recognized the value of working with and for the communities to ensure cultural sensitivity. We intend to use the lessons learned from this process to inform future co‐design for other ethno‐cultural groups. The dementia‐related resources are available in several languages at www.dementiawellness.

